# Ranking Features Distinguish Vestibular Migraine with Positional Vertigo from Benign Paroxysmal Positional Vertigo Using Machine Learning

**DOI:** 10.3390/diagnostics16213699

**Published:** 2026-11-09

**Authors:** Kaijun Xia, Linlin Wang, Xingqian Shen, Xiaoye Chen, Ziying Xu, Xuanyu Shi, Lu Wang, Hongchang Wang, Hongjun Xiao, Bo Liu, Yangming Leng

**Affiliations:** 1 Department of Otorhinolaryngology-Head and Neck Surgery, ENT Institute, Union Hospital, Tongji Medical College, Huazhong University of Science and Technology, Wuhan 430022, China; xiakaijun626@hust.edu.cn (K.X.); 2020xh5020@hust.edu.cn (L.W.); sxq15207158114@163.com (X.S.); d202672709@hust.edu.cn (X.C.); xzy15871263046@163.com (Z.X.); m202576456@hust.edu.cn (X.S.); m202576393@hust.edu.cn (L.W.); 18696159860@163.com (H.W.); xhjent_whxh@hust.edu.cn (H.X.); 2 Hubei Province Clinical Research Center for Deafness and Vertigo, Wuhan 430022, China

**Keywords:** machine learning, vestibular migraine, positional vertigo, benign paroxysmal positional vertigo

## Abstract

**Objectives**: Vestibular migraine with positional vertigo (VMwPV) can mimic benign paroxysmal positional vertigo (BPPV), leading to diagnostic challenges. This study aimed to compare clinical and nystagmus-related features between VMwPV and BPPV patients and to establish a differential diagnostic model using machine learning. **Methods**: 101 VMwPV and 219 BPPV patients were enrolled. The nystagmus-related features included spontaneous nystagmus (SN) and parameters during positional testing: latency, pure vertical or vertical-component positional nystagmus (PN), PN lasting <1 min, and the maximum slow phase velocity (SPV_max_) of PN. Vertigo/dizziness during positional testing was also included. Using multiple machine learning algorithms, diagnostic models were constructed, and the optimal model was selected. The Shapley Additive Explanations (SHAP) analysis was applied to rank the feature importance. **Results**: (1) Compared with BPPV, VMwPV patients were more likely to have SN and pure vertical/vertical-component PN but less likely to have latency, PN lasting <1 min, and provoked vertigo/dizziness; the SPV_max_ of PN was lower in VMwPV than in BPPV. (2) No statistically significant differences in area under the curve (AUC) were observed among the five models. Logistic Regression was selected as the optimal model based on its superior interpretability. (3) Vertigo/dizziness during positional testing and PN lasting <1 min were ranked as the top two most important features based on SHAP analysis. These findings should be interpreted with caution due to group selection and incorporation bias. **Conclusions**: Apart from the subjective provoked vertigo/dizziness, other objective variables such as PN lasting <1 min may have potential value in differentiating VMwPV and BPPV, which require multicenter studies and prospective external validation.

## 1. Introduction

Vestibular migraine (VM) is a common cause of vertigo/dizziness, characterized by recurrent episodes of vestibular symptoms accompanied by migraine features. The vestibular manifestations vary widely among individuals, and a single patient may experience more than one type of vertigo over the disease course, including spontaneous vertigo, positional vertigo, visually induced vertigo, and head motion-induced vertigo/dizziness [1,2]. Benign paroxysmal positional vertigo (BPPV) is characterized by paroxysms of vertigo and nystagmus triggered by head position changes relative to gravity. The mechanisms of BPPV include the canalithiasis theory and the cupulolithiasis theory [3]. VM and BPPV are common causes of positional vertigo [1,4]. Beh et al. found that approximately one-quarter of VM patients manifested with positional vertigo during the ictal phase [5]. These BPPV-like symptoms in vestibular migraine with positional vertigo (VMwPV) pose significant challenges to clinical differential diagnosis [6,7,8,9,10].

The mechanisms underlying positional vertigo and positional nystagmus (PN) in VMwPV patients remain unclear. A migraine episode could sensitize the vestibular nuclei to the extent that suppression from the cerebellar nodulus and uvula (NU) is no longer adequate to maintain stability, particularly when NU activity is modulated by changing head orientation, resulting in ictal episodes of vestibular dysfunction that are induced by head tilts (e.g., positional vertigo/nystagmus) [11]. The PN in VM patients is significantly correlated with lactate peaks in the occipital lobe, suggesting that occipital metabolic changes induced by cortical spreading depression may be one of the underlying pathophysiological mechanisms [12]. Migraine-related trigemino-vascular system activation may cause fluid extravasation in the inner ear, thereby altering the specific gravity between the endolymph and the cupula, producing PN and positional vertigo similar to those of a light/heavy cupula [13,14,15].

PN in VMwPV often lacks typical diagnostic features and may present with either peripheral and/or central patterns [16,17,18]. Although previous studies on this topic have involved relatively small cohorts [19,20], several key observations have emerged. El-Badry et al. summarized the characteristics of PN in VMwPV: pure vertical; neither latency nor fatigability; persistent and markedly suppressed by visual fixation; and could be cured by anti-migraine medications [21]. Several studies have already focused on the differences in PN between VM and BPPV. Young et al. found that, compared with the paroxysmal nystagmus of BPPV, VM patients exhibited persistent PN with significantly slower decay [22]. Similarly, by analyzing the temporal pattern of the slow-phase velocity (SPV) time profiles in 60 patients with positional vertigo and horizontal nystagmus during Roll test, Lechner et al. found that unlike the crescendo-decrescendo pattern and interaural asymmetry in horizontal semicircular canal canalolithiasis (HSC-BPPV-can), the temporal profile of SPV in most VMwPV patients was flat and symmetrical with either ear down [23].

Previous studies on distinguishing VMwPV from BPPV have primarily focused on individual or a limited number of nystagmus parameters using conventional statistical approaches, which may neglect the interplay among multiple features. Machine learning enables the simultaneous integration of diverse parameters and has shown utility in detecting patterns within large, unstructured, and complex datasets. Until now, no study has applied machine learning to compare the nystagmus features between VMwPV and BPPV patients. In the present study, we used multiple machine learning methods to compare VMwPV and BPPV in terms of vertigo/dizziness in positional testing and multiple objective parameters of nystagmus. Our findings may offer clinicians a practical basis for the differential diagnosis of positional vertigo and guide future research into the pathogenesis of VMwPV.

## 2. Participants and Methods

### 2.1. Participants

This study enrolled patients with positional vertigo who attended the Department of Otorhinolaryngology–Head and Neck Surgery at Union Hospital, Tongji Medical College, Huazhong University of Science and Technology, between May 2025 and June 2026 and were diagnosed as VM/probable VM or BPPV according to the Bárány Society criteria [1,24]. VMwPV was defined as a diagnosis of VM/probable VM in patients whose chief complaint was positional vertigo. VMwPV patients who experienced vertigo/dizziness during positional testing were defined as ictal-phase VMwPV patients, while those who did not experience vertigo/dizziness were defined as interictal VMwPV patients. All patients were independently evaluated by two experienced vestibular specialists. In diagnostically ambiguous cases, the two specialists discussed the case until a consensus was reached. Exclusion criteria were: (1) subjects without nystagmus during the Dix-hallpike/Roll Test; (2) final diagnosis other than BPPV or VM/probable VM; (3) secondary BPPV; (4) controversial syndromes as defined by the Bárány Society criteria [24]: multiple-canal BPPV, canalolithiasis of the anterior canal BPPV, cupulolithiasis of the posterior canal BPPV; and (5) cases with comorbid VM and BPPV.

A brain MRI or neurology consultation was recommended if patients presented with clinical features suggestive of a central vestibular disorder—such as vertigo attacks accompanied by disturbances of consciousness, visual symptoms (e.g., diplopia, visual field defects, or transient monocular visual loss), ataxia, limb numbness and weakness, hoarseness, dysphagia, or other focal neurological symptoms—or if they exhibited central oculomotor signs on videonystagmography (VNG) testing, including gaze-evoked nystagmus, saccadic dysmetria, saccadic pursuit, asymmetric optokinetic nystagmus, or pure vertical nystagmus. All patients discontinued medications known to affect nystagmus (including sedatives, vestibular suppressants, etc.), caffeine, and alcohol at least 48 h before VNG testing.

This study was conducted in compliance with the tenets of the Declaration of Helsinki. Informed consent was obtained from every participant. The project was approved by the ethical committee of Tongji Medical College of Huazhong University of Science and Technology (Institutional Review Board approval [2025] No. 0619).

### 2.2. VNG

All participants in the present study underwent vestibular tests by using an infrared videonystagmography system (Visual Eyes VNG, Micromedical Technologies, Chatham, IL, USA) in accordance with a standard test protocol, including the spontaneous nystagmus (SN) test, gaze test, saccades, smooth pursuit, optokinetic test, Dix-hallpike Test and Roll Test [3].

### 2.3. Data Collection

The clinical variables included gender, age, history of migraine, photophobia, and phonophobia. The variables for machine learning collected during vestibular tests are summarized in Table 1. SPV was automatically measured by the VNG software. When both horizontal and vertical components were present, each component was measured separately, and the larger value was taken as the maximal SPV (SPV_max_) of PN. Pure vertical PN was defined as nystagmus with a vertical component only, without horizontal or torsional components, while vertical-component PN was defined as nystagmus with both a vertical and a horizontal component, but without the typical torsional component. Vertical nystagmus with a torsional component is highly typical in PSC-BPPV but is extremely rare in VMwPV. Including it as an independent variable could readily lead to overfitting and distortion of feature importance assessment in the machine learning models. Therefore, with regard to the direction of nystagmus, we did not include the presence of vertical nystagmus with a torsional component as a separate variable in our model. For pure vertical or vertical-component PN, the SPV_max_ of the vertical component alone was calculated and used for further analysis. The SPV_max_ of SN was defined as the maximal SPV of either the vertical or horizontal component. The differential diagnosis between SN and pseudo-spontaneous nystagmus (PSN) is achieved according to the study of Asprella-Libonati et al. [25].

All patients underwent a predefined VNG protocol. The examiners who performed and recorded the VNG findings were blinded to the final diagnosis. The investigators who retrospectively extracted the six model variables from standardized VNG reports and clinical records were blinded to the diagnostic adjudication. The clinicians who established the final diagnosis were blinded to the specific combination of variables subsequently entered into the machine-learning models.

### 2.4. Statistical Analysis

All categorical variables were encoded as binary variables, while SPV_max_ and age were retained as continuous variables. A two-stage analytical strategy was adopted in this study.

Stage 1: Univariate intergroup comparisons. To comprehensively describe the differences between the VMwPV and BPPV groups, univariate analyses were performed on all 11 independent variables. Continuous variables were expressed as mean ± standard deviation or median (interquartile range, IQR). Categorical variables and grade variables were expressed as frequency (percentage). A Shapiro–Wilk test was used to test the normality of data. Normally distributed continuous variables were compared using the independent-samples t test, whereas non-normally distributed continuous variables were compared using the Mann–Whitney U test. Categorical variables were compared using the chi-square test or Fisher’s exact test for two independent samples. Statistical significance was set at *p* < 0.05.

Stage 2: Machine learning analysis. To focus on the discriminative value of variables during vestibular tests for differentiating VMwPV from BPPV, only characteristics in Table 1 were included in machine learning. To further evaluate whether the model’s discriminative ability depends on a specific BPPV phenotype, we performed sensitivity analyses comparing VMwPV with PSC-BPPV and VMwPV with HSC-BPPV (canalolithiasis and cupulolithiasis), using the same repeated stratified 5-fold cross-validation (10 repeats) and Logistic Regression.

### 2.5. Multi-Model Development and Hyperparameter Settings

Model implementation and training were conducted using R4.2.3. Various machine learning models were used, including Logistic regression, Lasso Regression [26], Random forest [27], XGBoost [28], and Support Vector Machine [29]. The rationale for the selection of these models is covered in Table 2. Hyperparameter tuning was performed within a nested cross-validation framework. The outer loop consisted of repeated stratified 5-fold cross-validation with 10 repeats (50 outer validation folds) for performance evaluation. Within each outer training set, an independent inner 5-fold cross-validation was performed to select the optimal hyperparameters. The outer validation folds were never used for hyperparameter selection. For each model, a predefined tuning grid was evaluated: Logistic Regression: No hyperparameters were tuned. Lasso Regression: alpha = 1, and lambda was searched over 20 values on a logarithmic scale from 1 × 10^−4^ to 1 × 10^−1^. Random Forest: mtry was tuned using tuneLength = 5 (candidate values: 2, 3, 4, 5, 6). XGBoost: tuneLength = 5, searching over nrounds, max_depth, eta, gamma, colsample_bytree, min_child_weight, and subsample. Support Vector Machine: A full 6 × 6 grid was used, with sigma ranging from 10^−3^ to 10^0^ (6 values) and C ranging from 2^−2^ to 2^4^ (6 values). The optimal hyperparameter combination for each model was defined as the one that yielded the highest mean AUC across the inner cross-validation folds. The optimal hyperparameters selected for the final models were as follows: Lasso Regression, lambda = 0.0336; Random Forest, mtry = 2; XGBoost, nrounds = 50, max_depth = 1, eta = 0.4, gamma = 0, colsample_bytree = 0.6, min_child_weight = 1, subsample = 0.5; Support Vector Machine, sigma = 0.001, C = 6.964. To further address the non-independence of the resampling folds, all 95% confidence intervals for the performance metrics were calculated using a subject-level Bootstrap approach (2000 resamples). The tuning curves for all models are shown in Supplementary Figure S1.

### 2.6. Model Evaluation and Interpretability

Pairwise comparisons of area under the curve (AUC) between models were performed using DeLong’s test for paired receiver operating characteristic (ROC) curves. To account for multiple comparisons, Benjamini–Hochberg false discovery rate (FDR) correction was applied. A two-sided FDR-adjusted *p*-value < 0.05 was considered statistically significant. In addition, accuracy, sensitivity, specificity, positive predictive value (PPV), negative predictive value (NPV), and F1-score were also analyzed [30]. Calibration was assessed with the Brier score and calibration curves. Clinical usefulness was assessed with decision curve analysis across threshold probabilities of 0–0.99. Accuracy, sensitivity, specificity, PPV, NPV, and F1-score were all calculated using a fixed probability threshold of 0.5. That is, a patient was classified as VMwPV if the predicted probability was greater than 0.5 and as BPPV otherwise. To enhance model interpretability, SHAP was applied to the five final models trained on the full dataset after repeated stratified 5-fold cross-validation with nested hyperparameter tuning. SHAP values were computed using the fastshap package (nsim = 50), and beeswarm plots were generated to show feature contribution direction and importance ranking.

## 3. Results

### 3.1. Clinical Features of VMwPV and BPPV

**Figure 1:**
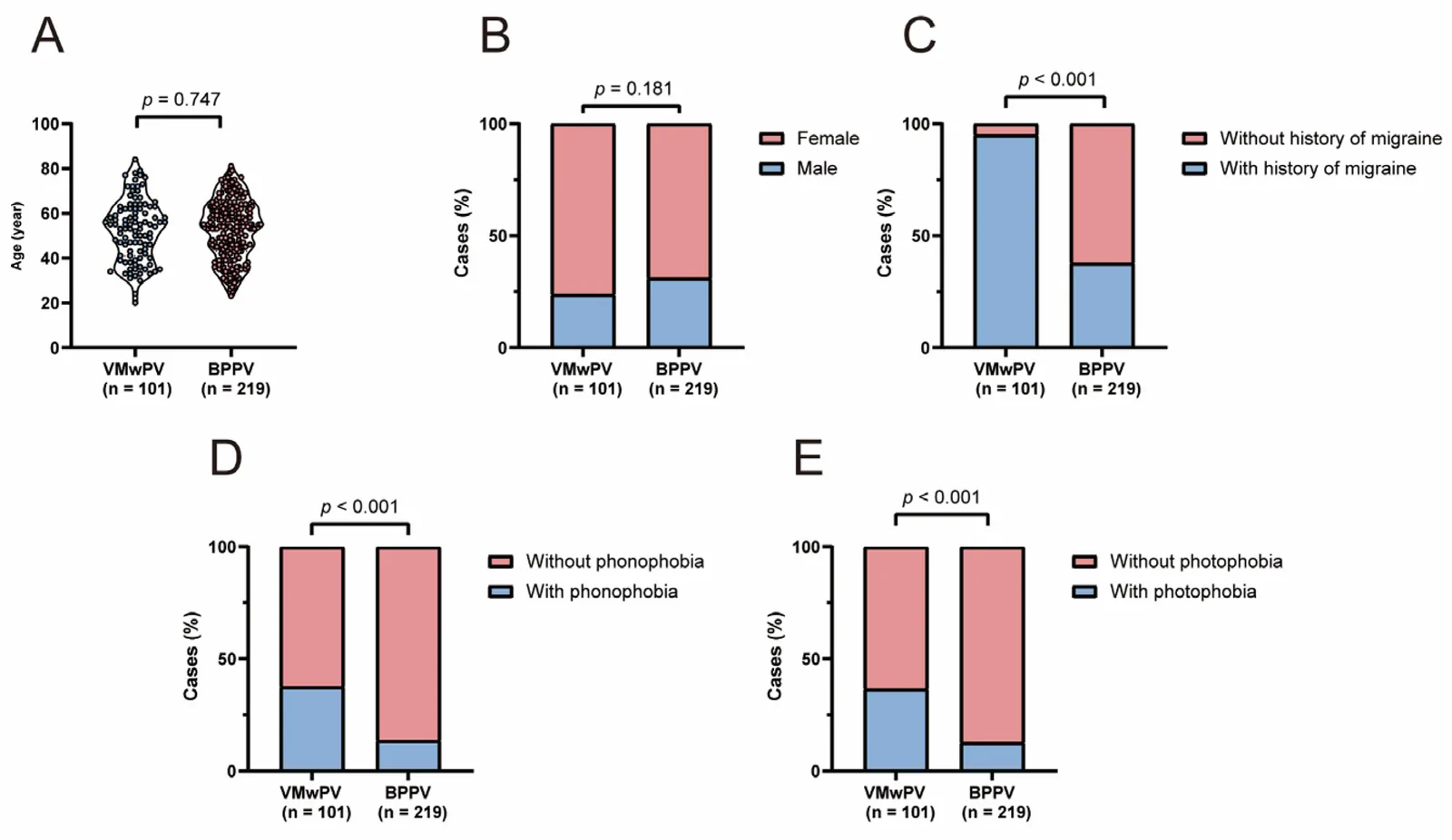
Comparison of the clinical features between VMwPV group and BPPV group in (**A**) Age, (**B**) Gender, the prevalence of (**C**) migraine history, (**D**) phonophobia and (**E**) photophobia. VMwPV, vestibular migraine with positional vertigo; BPPV, benign paroxysmal positional vertigo.

In the present study, we enrolled 101 VMwPV patients, including 34 VM cases and 67 probable VM cases. Additionally, 219 BPPV patients were included, comprising 159 patients with posterior semicircular canal BPPV (PSC-BPPV), 50 patients with HSC-BPPV-can and 10 patients with horizontal semicircular canal cupulolithiasis (HSC-BPPV-cup). The male/female ratio in VMwPV group and BPPV group were 24/77 and 68/151, with no significant difference (χ^2^ = 1.792, *p* = 0.181). The age in VMwPV group and BPPV group was 54(41, 62) and 54(43, 63), and no significant difference was found (Z = −0.323, *p* = 0.747). The VMwPV group had a significantly higher proportion of migraine history (95% vs. 37.9%), photophobia (36.6% vs. 12.8%), and phonophobia (37.6% vs. 13.7%) compared with the BPPV group (all *p* < 0.001) (Figure 1).

### 3.2. Comparison of the Presence of Vertigo in Positional Testing and Features of Nystagmus Between VMwPV and BPPV

**Figure 2:**
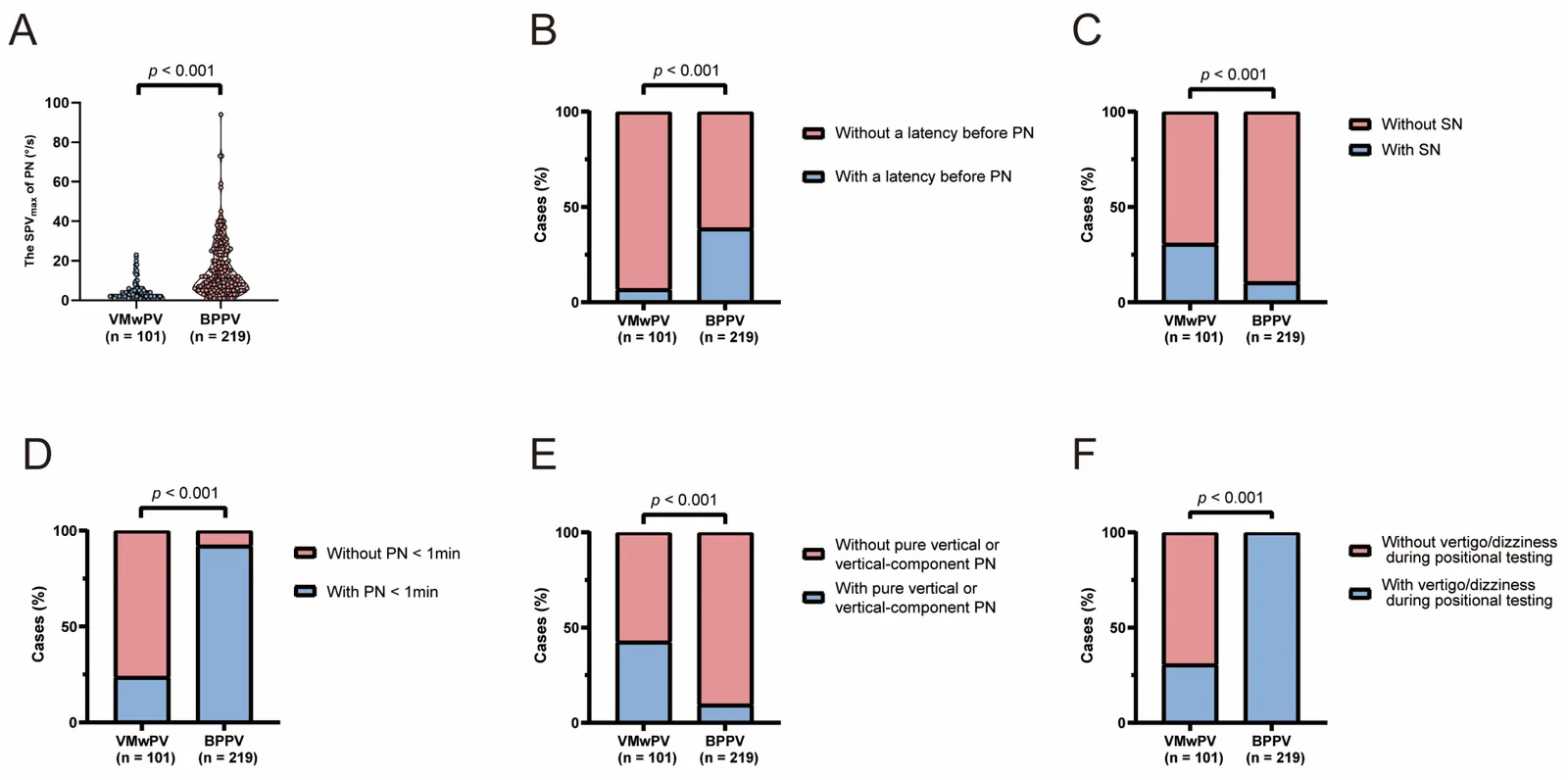
Comparison of the nystagmus features between VMwPV group and BPPV group in (**A**) the SPV_max_ of PN, the prevalence of (**B**) a latency before PN, (**C**) spontaneous nystagmus, (**D**) PN lasting <1 min, (**E**) pure vertical or vertical-component PN, and (**F**) vertigo/dizziness during positional testing. VMwPV, vestibular migraine with positional vertigo; BPPV, benign paroxysmal positional vertigo; PN, positional nystagmus; SN, spontaneous nystagmus; SPV_max_, maximum slow phase velocity.

As shown in Figure 2, the median SPV_max_ of PN were 3 (2, 5) °/s in the VMwPV group and 11(6,23) °/s in the BPPV group. The former was significantly lower than the latter (Z = −9.440, *p* < 0.001). The prevalence of SN in the VMwPV group (30.7%) was significantly higher than in the BPPV group (10.5%) (χ^2^ = 20.088, *p* < 0.001). The latency was observed in 7 (6.9%) VMwPV patients and 85(38.8%) BPPV patients, which was significantly lower in the VMwPV group than in the BPPV group (χ^2^ = 34.299, *p* < 0.001). PN lasting < 1 min was present in 24(23.8%) VMwPV patients and 202(92.2%) BPPV patients. The prevalence of this phenomenon in the VMwPV group was significantly lower than in the BPPV group (χ^2^ = 156.223, *p* < 0.001). As for pure vertical or vertical-component PN during positional testing, it was observed in 43(42.6%) VMwPV patients and 21(9.6%) BPPV patients and was more frequent in the VMwPV group than in the BPPV group (χ^2^ = 47.004, *p* < 0.001). Positional vertigo/dizziness was provoked in 31(30.7%) VMwPV patients and all BPPV patients, which was less frequent in the VMwPV group than in the BPPV group (χ^2^ = 194.281, *p* < 0.001).

Among the 31 patients with VMwPV who exhibited SN, the directions of SN were diverse: 4 cases showed upbeat nystagmus, 4 showed downbeat nystagmus, 4 showed nystagmus with a vertical-component, and 19 showed horizontal nystagmus. The directions of SN in all 23 BPPV patients were horizontal, comprising 13 cases of PSC-BPPV, 7 cases of HSC-BPPV-can, and 3 cases of HSC-BPPV-cup. As for HSC-BPPV-can, PSN was seen in 5 cases of HSC-BPPV-can, while true SN was seen in 2 cases of HSC-BPPV-can. As for the HSC-BPPV-cup, PSN was seen in 2 cases of HSC-BPPV-cup, while true SN was seen in 1 case of HSC-BPPV-cup. The SPV_max_ of SN in the VMwPV group and the BPPV group were 2(1,2) °/s and 2(1,2) °/s, respectively. And no significant difference was found (Z = −0.197, *p* = 0.843). The intensity of pure vertical or vertical-component PN in VMwPV group and BPPV group were 3(2,5) °/s and 3(2, 4) °/s, without significant difference (Z = −0.689, *p* = 0.491).

### 3.3. Machine Learning Models

As shown in Table 3, the mean (95%CI) of AUC in Logistic regression, Lasso Regression, Random Forest, XGBoost, and Support Vector Machine were 0.9426 (0.9053–0.9742), 0.9405 (0.9009–0.9741), 0.9371 (0.8996–0.9690), 0.9427 (0.9047–0.9745), and 0.9392 (0.9001–0.9716), respectively. Because no statistically significant differences in AUC were observed among the five models (DeLong test, all FDR-adjusted *p* > 0.05), we selected Logistic Regression as the representative model for detailed interpretation. This choice was based on its superior interpretability, as the regression coefficients can be directly translated into odds ratios, and its ease of clinical deployment.

**Figure 3:**
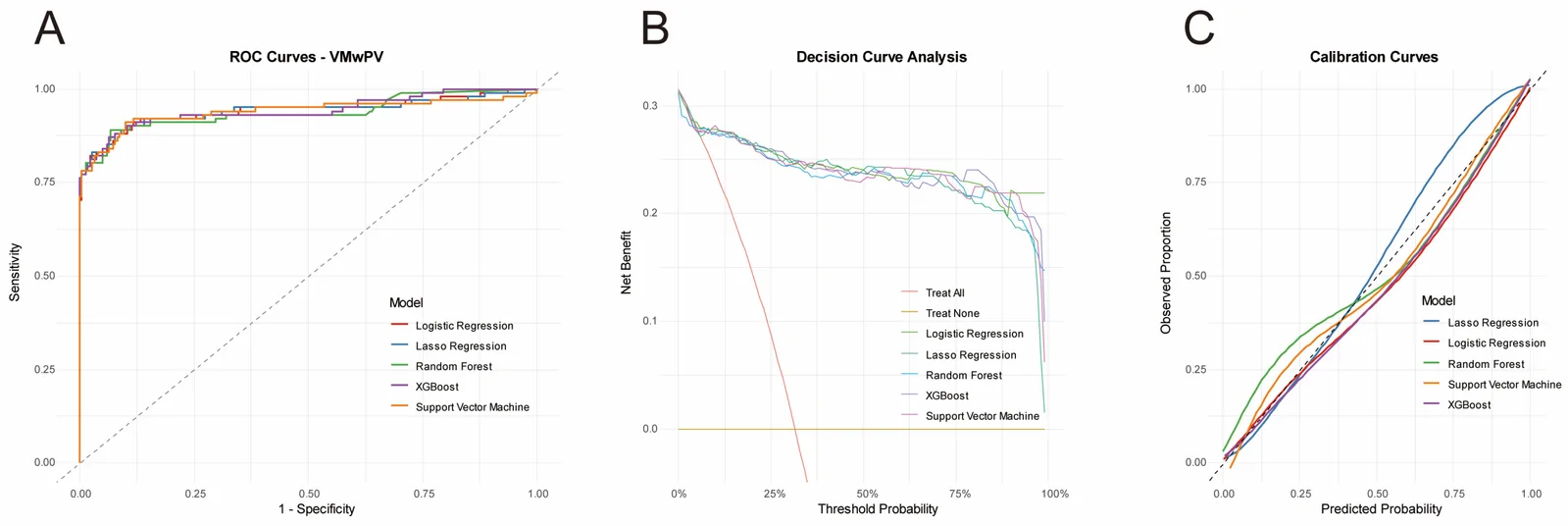
The performance of all models. The ROC curve of Logistic regression, Lasso Regression, Random Forest XGBoost and Support Vector Machine (**A**), the decision curve analysis (**B**), and the calibration curves (**C**) of all models.

Model calibration was assessed using the Brier score and calibration plots. Brier scores were 0.0592 for Logistic Regression, 0.0605 for Lasso Regression, 0.0640 for Random Forest, 0.0618 for XGBoost, and 0.0612 for Support Vector Machine, all below 0.1, indicating excellent calibration across all five models. Calibration curves showed that the predicted probabilities of all models were close to the ideal diagonal line (y = x) across the range of 0 to 1, indicating good agreement between predicted and observed probabilities (Figure 3C). Decision curve analysis showed that all models provided higher net benefit than the “treat all” and “treat none” strategies, suggesting meaningful clinical utility (Figure 3B).

As shown in Supplementary Material S1, the model showed good discriminative performance in both comparing VMwPV with PSC-BPPV and VMwPV with HSC-BPPV. The AUC of sensitivity analyses comparing VMwPV with PSC-BPPV was 0.9365 (0.8918–0.9717), and the AUC of sensitivity analyses comparing VMwPV with HSC-BPPV was 0.9554 (0.9231–0.9811). The performance was comparable across subgroups, suggesting that the model’s discriminative ability was not driven by a single BPPV subtype.

### 3.4. The Feature Importance

**Figure 4:**
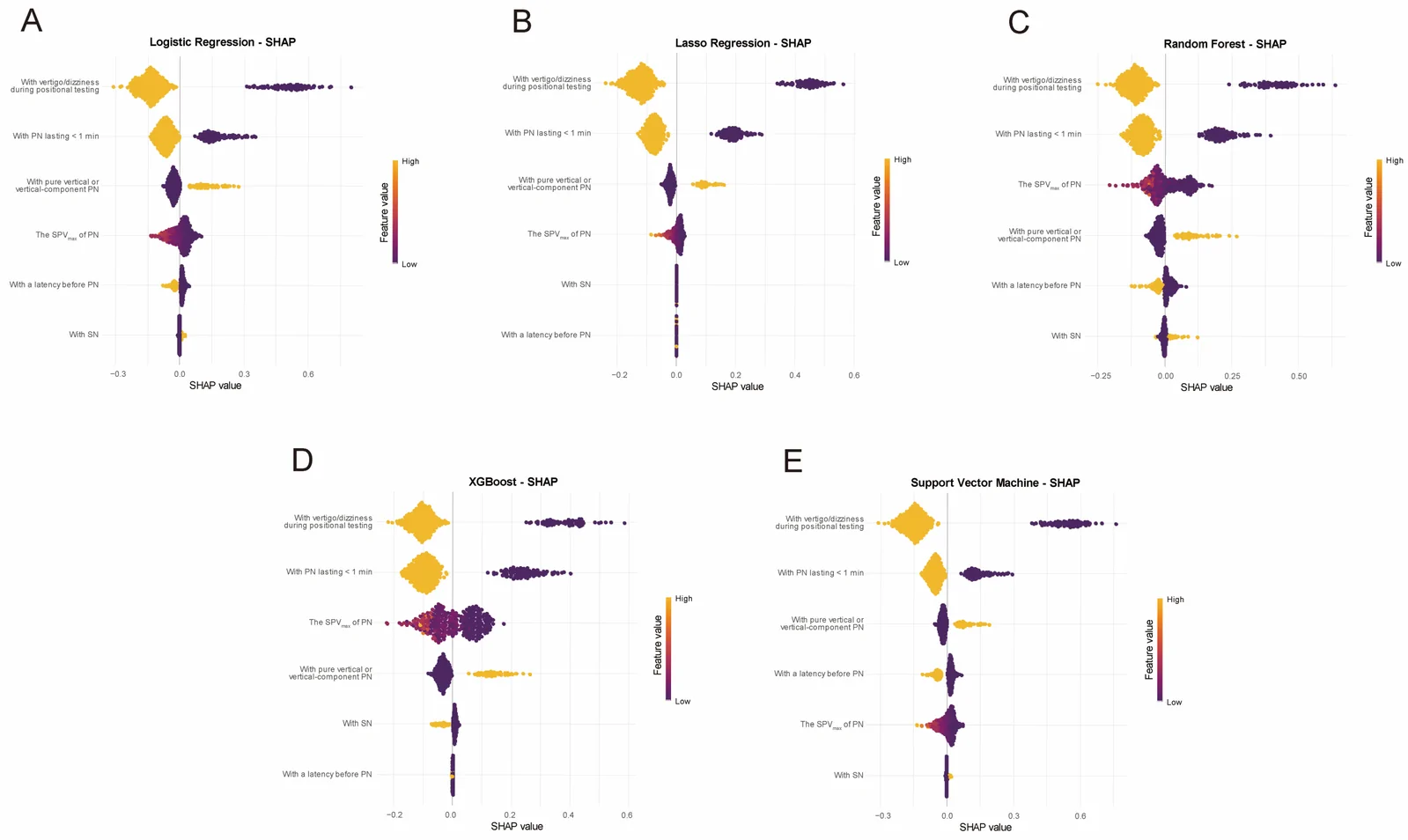
SHAP feature importance beeswarm plots of five machine learning models for distinguishing VMwPV from BPPV: (**A**) Logistic Regression, (**B**) Lasso Regression, (**C**) Random Forest, (**D**) XGBoost, and (**E**) Support Vector Machine. Color from purple to yellow indicates the corresponding feature value from low to high (e.g., higher SPV_max_ is shown in yellow); x-axis (SHAP value) represents the direction and magnitude of each feature’s contribution to the model’s prediction of “VMwPV” (positive values favor the diagnosis of VMwPV, and negative values favor BPPV). y-axis lists features in descending order of mean absolute SHAP values, with the top-ranked features being the most influential predictors across models. VMwPV, vestibular migraine with positional vertigo; BPPV, benign paroxysmal positional vertigo; PN, positional nystagmus; SN, spontaneous nystagmus; SPV_max_, maximum slow phase velocity; SHAP, Shapley Additive Explanations.

SHAP analyses were performed for all five models, and the results are shown in Figure 4. The SHAP-based feature importance rankings were largely consistent across models, with vertigo/dizziness during positional testing and PN lasting <1 min consistently ranking as the most influential predictors.

### 3.5. Sensitivity Analyses

Since provoked vertigo/dizziness was a necessary requirement for BPPV diagnosis, we further removed “vertigo/dizziness during positional testing” and reran all analyses using the same repeated stratified 5-fold cross-validation (10 repeats) framework (Supplementary Material S2). The mean AUC decreased from 0.9426 to 0.9350 for Logistic Regression, from 0.9405 to 0.9326 for Lasso Regression, from 0.9371 to 0.9235 for Random Forest, from 0.9427 to 0.9313 for XGBoost, and from 0.9392 to 0.9225 for Support Vector Machine. After removing “vertigo/dizziness during positional testing”, the DeLong comparison showed that Logistic Regression and Lasso Regression had significantly higher AUC than Support Vector Machine (FDR-adjusted *p* = 0.02 and 0.03, respectively), whereas no other pairwise comparisons reached statistical significance. In the SHAP analysis, PN lasting <1 min remained the leading predictor, indicating that the models learned independent nystagmus features beyond “vertigo/dizziness during positional testing”.

In addition, we further constructed model analysis using three features that are independent of the Bárány Society BPPV diagnostic criteria: SN, SPV_max_ of PN, and pure vertical/vertical-component PN. We repeated the same nested cross-validation framework (repeated stratified 5-fold CV, 10 repeats) to evaluate its performance. Although the sensitivity has slightly decreased, the AUCs of these models still demonstrated substantial and statistically significant discriminative ability (AUC > 0.83) (Supplementary Material S3). The DeLong comparison showed that Support Vector Machine had a significantly lower AUC compared to Logistic Regression, Lasso Regression, and XGBoost (all FDR-adjusted *p* < 0.05). As for the SHAP analysis, the feature importance ranking among the three objective variables was largely consistent across models, with SPV_max_ of PN and pure vertical or vertical-component PN ranking as the top two predictors.

### 3.6. Subgroup Analyses Within the VMwPV Cohort

**Figure 5:**
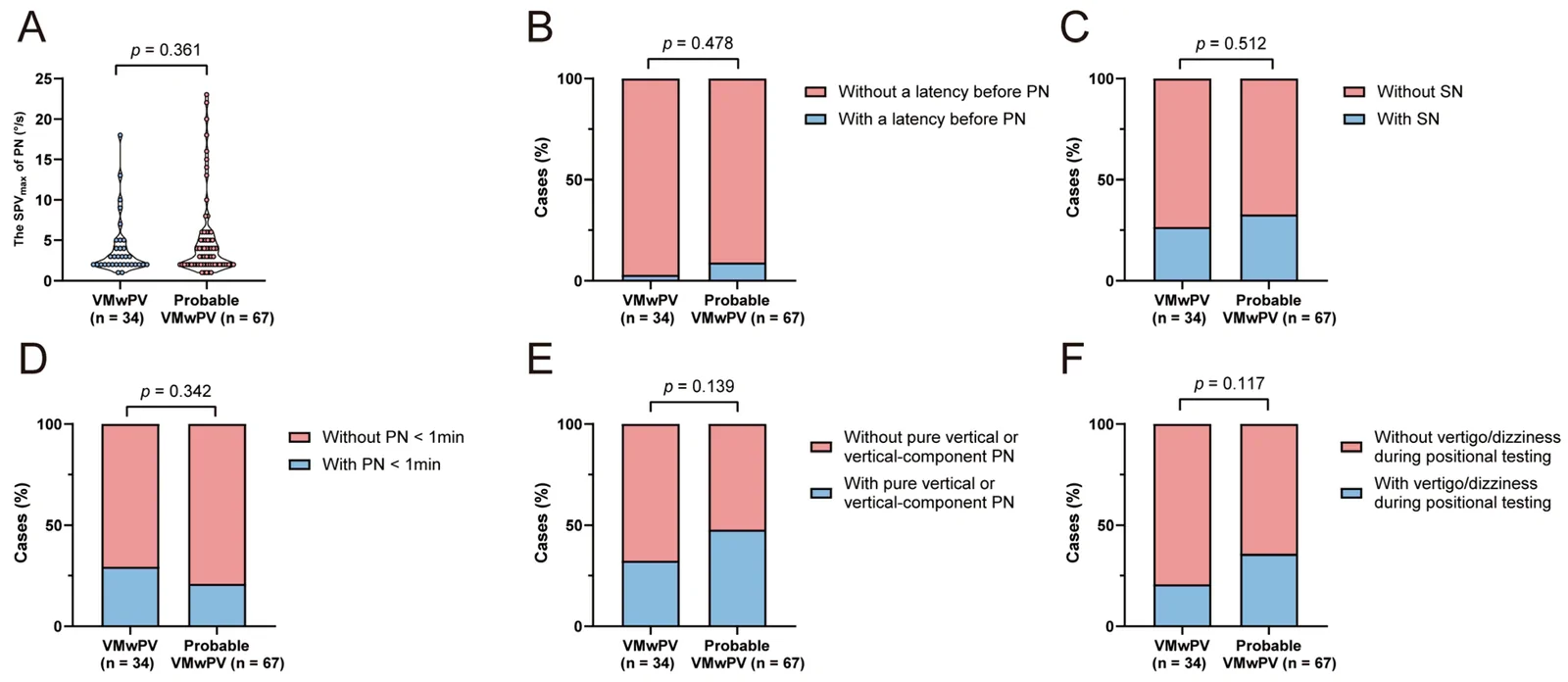
Comparison between VMwPV and probable VMwPV patients in (**A**) the SPVmax of PN, the prevalence of (**B**) a latency before PN, (**C**) SN, (**D**) PN < 1 min, (**E**) pure vertical or vertical-component PN, and (**F**) vertigo/dizziness during positional testing. VMwPV, vestibular migraine with positional vertigo; PN, positional nystagmus; SN, spontaneous nystagmus; SPV_max_, maximum slow phase velocity.

We further analyzed the nystagmus characteristic between VMwPV and probable VMwPV. The SPV_max_ of PN in VMwPV and probable VMwPV were 2.5(2, 4.25) °/s and 3(2, 5) °/s (Z = −0.913, *p* = 0.361). The latency was observed in 1(2.9%) VMwPV cases and 6(9.0%) probable VMwPV cases (χ^2^ = 1.265, *p* = 0.478). The prevalence of SN was observed in 9(26.5%) VMwPV cases and 22(32.8%) cases (χ^2^ = 0.430, *p* = 0.512). PN lasting <1 min was present in 10(29.4%) VMwPV patients and 14(20.9%) probable VMwPV patients (χ^2^ = 0.903, *p* = 0.342). Pure vertical or vertical-component PN was seen in 11(32.4%) VMwPV cases and 32(47.8%) probable VMwPV (χ^2^ = 2.190, *p* = 0.139). Vertigo/dizziness was provoked in 7(20.6%) VMwPV cases and 24(35.8%) probable VMwPV cases (χ^2^ = 2.460, *p* = 0.117). No significant difference was observed in all these nystagmus features between VMwPV and probable VMwPV (Figure 5). These results support the homogeneity of the two subgroups with respect to the variables used in the models.

**Figure 6:**
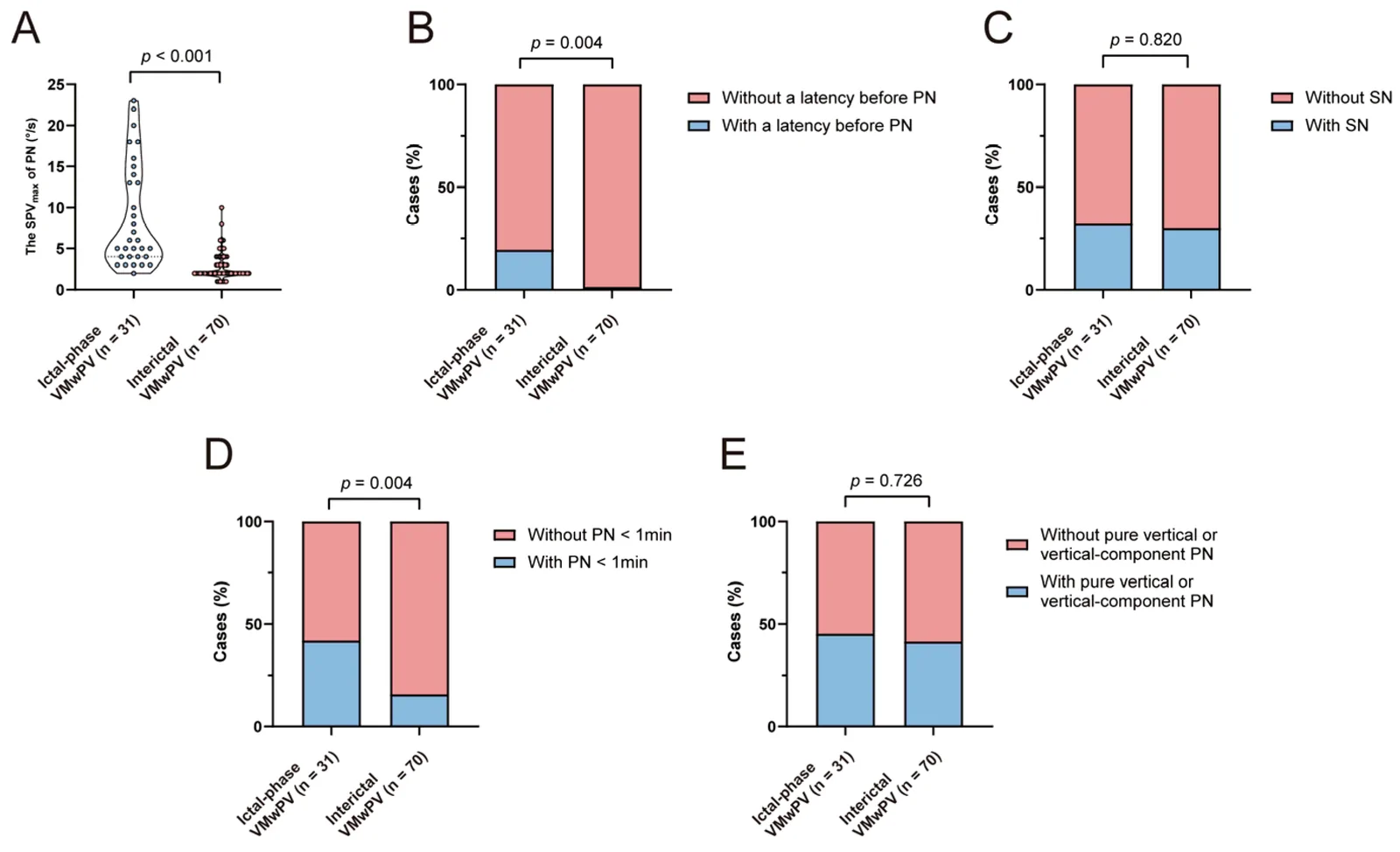
Comparison of the nystagmus characteristics between VMwPV patients of ictal and interictal phases in (**A**) the SPV_max_ of PN, the prevalence of (**B**) a latency before PN, (**C**) SN, (**D**) PN < 1 min, and (**E**) pure vertical or vertical-component PN. VMwPV, vestibular migraine with positional vertigo; PN, positional nystagmus; SN, spontaneous nystagmus; SPV_max_, maximum slow phase velocity.

In addition, nystagmus characteristics were compared between ictal (those with vertigo/dizziness during positional testing) and interictal phases (those without vertigo/dizziness during positional testing). The SPV_max_ of PN in ictal and interictal phases was 6(4,14) and 2(2,3.25) °/s (Z = −6.365, *p* < 0.001). The latency was observed in 6(19.4%) ictal-phase VMwPV and 1(1.4%) interictal VMwPV cases (χ^2^ = 10.704, *p* = 0.004). The prevalence of SN was observed in 10(32.3%) ictal-phase VMwPV and 21(30.0%) interictal VMwPV cases (χ^2^ = 0.051, *p* = 0.820). PN lasting <1 min was present in 13(41.9%) ictal-phase VMwPV patients and 11(15.7%) interictal VMwPV cases (χ^2^ = 8.154, *p* = 0.004). Pure vertical or vertical-component PN was seen in 14(45.2%) ictal-phase VMwPV patients and 29(41.4%) interictal VMwPV cases (χ^2^ = 0.122, *p* = 0.726). Compared with those in the ictal phase, cases in interictal phases showed weaker intensity of PN, lower prevalence of latency, and lower prevalence of PN <1 min. No significant difference was observed in the prevalence of SN and pure vertical or vertical-component PN (Figure 6).

## 4. Discussion

To analyze the subjective symptom (vertigo or dizziness) in positional testing and multiple objective nystagmus features in VMwPV and BPPV patients, five machine learning approaches were employed to establish differential diagnostic models. Because no statistically significant differences in AUC were observed among the five models, we selected Logistic Regression as the optimal model based on its superior interpretability. SHAP analysis was further applied to rank the above features, enabling visualization of the importance of each feature in differentiating VMwPV from BPPV. In the optimal model, the top two most important features were the presence of provoked vertigo/dizziness and PN lasting <1 min.

Previous studies typically examined individual or a limited number of nystagmus parameters in VM patients using conventional statistical approaches. Although such analyses are useful for identifying associations between individual features and disease groups, they may not fully characterize the joint information contained in multidimensional nystagmus features. In the present study, multiple machine learning models were employed to integrate both a subjective feature and a range of objective nystagmus features from patients with VMwPV and BPPV. This approach enables simultaneous modeling of multiple features and can accommodate potentially nonlinear relationships and interactions. Furthermore, SHAP analysis was used to interpret the models by quantifying the contribution of each feature to model output, allowing the information learned by the models to be examined at the feature level.

### 4.1. The Clinical Characteristics Between VMwPV and BPPV

Our results showed no significant differences in gender or age between patients with VMwPV and BPPV. In the study by Mok et al., VM and BPPV patients showed no significant difference in gender distribution, but VM patients were younger than those with BPPV [7]. This discrepancy may be attributed to the prolonged course and repetitive bouts of VMwPV, as disease duration and attack frequency may influence the age of consultation.

In contrast to the similarities in demographic characteristics, migraine history, phonophobia, and photophobia were more frequently observed in patients with VMwPV. It is consistent with the study by Chen et al. [8], which demonstrated that headache in patients with positional vertigo had a strong predictive value for VMwPV. In contrast, Mok et al. reported no significant differences between VM and BPPV patients in prevalence of migraine history, phonophobia, and photophobia [7]. One possible explanation is the cohort composition, as Mok et al. included VM patients with both spontaneous and positional vertigo, whereas our cohort was restricted to VMwPV patients. These findings suggest that VM patients with different vestibular symptom patterns (spontaneous versus positional vertigo) may have distinct accompanying symptom profiles, and future studies should further perform subgroup analyses based on vestibular phenotypes (e.g., spontaneous or provoked vertigo).

### 4.2. The Presence of Vertigo/Dizziness During Positional Testing in VMwPV and BPPV

SHAP analysis identified the presence of provoked vertigo/dizziness as the most important feature for differentiating VMwPV from BPPV. An important finding underlying this contribution was the apparent dissociation between PN and subjective vertigo/dizziness in VMwPV. In the present study, fewer VMwPV patients (30.7%) experienced vertigo/dizziness during positional testing than BPPV patients (100%). Previous studies found that 11–55.4% of VM patients exhibited interictal PN without vertigo [31,32]. These findings, together with our results, indicate that VMwPV should be considered in case of PN without associated vertigo/dizziness during positional testing.

### 4.3. The Nystagmus Features in VMwPV and BPPV

#### 4.3.1. The Duration of PN in VMwPV and BPPV

SHAP analysis identified PN lasting <1 min as the second most important feature for differentiating VMwPV from BPPV. Previous studies reported persistent PN in VM patients [15,22,33]. Persistent horizontal nystagmus during Roll test was also reported in VMwPV patients [23]. Our results are generally consistent with the above findings, suggesting that the duration of PN may serve as an important differential diagnostic indicator when distinguishing VMwPV from BPPV, particularly canalolithiasis.

#### 4.3.2. The SPV_max_ of PN in VMwPV and BPPV

In this study, compared with BPPV patients, VMwPV patients showed lower SPV_max_ of PN. Previous studies have consistently described relatively low-velocity PN in VM patients. In Lechner et al.’s study, the SPV during Roll test in HSC-BPPV-can was higher than that in VMwPV patients in both ear-down positions [23]. Polensek et al. retrospectively reviewed the nystagmus features of 26 VM patients and found that 89% of patients had low-velocity PN (2–7°/s) [34]. Our results are generally consistent with the above studies and showed that the intensity of PN carries relevant information for distinguishing VMwPV from BPPV. Moreover, nystagmus intensity in VM may not represent a stable trait. Previous studies have suggested that the intensity of nystagmus in VM patients was higher in the ictal phase than in the interictal phase [31], indicating varying nystagmus features depending on disease activity in VMwPV.

#### 4.3.3. The Pure Vertical or Vertical-Component PN in VMwPV and BPPV

In this study, compared with BPPV patients, VMwPV patients (42.6%) had a higher proportion of pure vertical or vertical-component PN. This figure was significantly higher than that reported by Polensek et al. (19%) [34]. This discrepancy may be attributed to the inclusion of both pure vertical and vertical-component nystagmus in the present study, whereas in Polensek et al.’s study, only pure vertical nystagmus was analyzed. El-Badry et al. reported that all 13 VMwPV patients exhibited vertical PN [21]. The inconsistency between their results and ours may be due to differences in sample size.

In the present study, 9.6% of BPPV patients also exhibited low-intensity pure vertical or vertical-component PN, with an intensity of 3 (2, 4) °/s. Previous studies have reported PN in healthy subjects [35,36,37,38]. The intensity of the vertical nystagmus in our BPPV patients fell well below the upper limit of 7°/s for vertical nystagmus in healthy subjects [39], suggesting that it may be a non-specific finding similar to the low-velocity PN occasionally observed in healthy individuals, rather than a distinct pathological mechanism. Its mechanism remains uncertain and may involve gravity effects on the otolith organs or subclinical vestibular abnormalities [37].

#### 4.3.4. The Latency of PN in VMwPV and BPPV

Latency may be a characteristic distinguishing the PN patterns of VMwPV and BPPV. Our study found that, compared with BPPV patients (38.8%), VMwPV patients had a lower incidence of latency before PN (6.9%), which is consistent with that reported by El-Badry et al., i.e., absence of latency in all VMwPV patients [21]. Polensek et al. found that 20(76%) VM patients had no latency [34]. The incidence of latency in Polensek et al.’ study was higher than our result, which may be due to the larger sample size and different inclusion criteria in our study. In the study by Yu et al., using the multiaxial repositioning chair, 187/285 (65.6%) patients with PSC-BPPV had latency [40], which is higher than the 39.9% of BPPV patients with latency in our study (using manual maneuvers). The latency preceding PN in BPPV could be influenced by the location of the detached otoconia. It has been reported that PSC-BPPV typically has a latency of one or few seconds, whereas HSC-BPPV (especially cupulolithiasis) often has a brief latency or no latency [24]. Furthermore, this difference may also be attributed to variations in positional testing methods, which may result in different accelerations during testing [41,42].

#### 4.3.5. The SN in VMwPV and BPPV

SN was another nystagmus characteristic that differed between VMwPV and BPPV in the present study. In the present study, a higher proportion of VMwPV patients presented with SN compared with BPPV patients. To date, no study has compared the prevalence of SN between VMwPV and BPPV. In the study by Waissbluth et al., 13/60 (21.7%) VM patients exhibited SN [43], which was similar to that in the present study (30.7%). Young et al. found a difference in SN occurrence during the ictal phase (71.3%) and the interictal phase (14.9%) in patients with VM [31]. The proportion of VMwPV patients with SN in the present study fell within the range reported above.

Various directions of SN exist in our VMwPV cohort, including pure vertical SN, vertical-component SN, and horizontal SN. Previous studies also reported the various directions of SN in VM [34,44,45]. Young et al. observed the SN in 44/67 (65.7%) VM patients, of whom there were horizontal and vertical SN [22]. Furthermore, a previous study suggested that the direction of SN may change in the same VM patient [31]. The precise mechanisms underlying variable direction of SN in VM patients remain unclear, and both peripheral and central vestibular dysfunction may be involved. Decompensated peripheral vestibular damage and damage to the ventral tegmental tract or the flocculus may cause horizontal SN and vertical SN, respectively [46,47]. However, whether this mechanism can account for horizontal and vertical SN in VMwPV patients remains to be verified.

The directions of SN in all BPPV patients were horizontal in the present study. Previous studies have found the presence of PSN in patients with HSC-BPPV-can and HSC-BPPV-cup [25,48,49,50,51,52,53]. SN has also been reported in approximately 26% of patients with PSC-BPPV [54].

### 4.4. Strengthens and Limitations

Compared with previous studies on nystagmus characteristics in VMwPV, the present study has several advantages. First, multiple machine learning algorithms with different learning mechanisms were compared, and SHAP analysis was further applied to improve model interpretability by quantifying the contribution of individual features to model predictions. Second, the presence of vertigo in positional testing and multiple objective parameters of nystagmus are readily obtainable, making our models more operationally feasible. In addition, our study also benefits from a relatively large sample size (101 VMwPV patients), and the models were established based on real-world clinical data rather than database-derived cohorts.

This study also has several limitations. First, this was a retrospective cross-sectional study and did not include long-term follow-up data from patients with positional vertigo. Second, since only a minority of VMwPV patients underwent additional vestibular function tests (e.g., video head impulse test and caloric test) and brain MRI, these data were not analyzed in the present study. Third, VM are highly heterogeneous in nature, with substantial fluctuations in clinical and functional profiles among subjects even within a single patient during the ictal and interictal phases, which may affect nystagmus features. Fourth, the interval between the most recent vertigo/dizziness attack and the VNG examination was not systematically documented for VMwPV patients. Moreover, since some controversial types of BPPV were excluded in the present study, our findings should be interpreted only within the BPPV phenotypes actually studied (PSC-BPPV and HSC-BPPV). Finally, this was a single-center exploratory study, which may limit the generalizability of our results. Future multicenter studies with prospective external validation in patients with undifferentiated positional vertigo are needed to optimize and validate the models.

## 5. Conclusions

This retrospective study employed machine learning methods to integrate objective nystagmus features and provoked vertigo/dizziness during positional testing in patients diagnosed with VMwPV or BPPV (PSC-BPPV and LSC-BPPV). While provoked vertigo/dizziness and PN lasting <1 min were identified as the top features, the high importance of provoked vertigo likely reflects incorporation bias, as it is a core diagnostic criterion for BPPV. Objective features such as PN lasting <1 min may have potential reference value. Multicenter studies and prospective external validation in patients with undifferentiated positional vertigo are needed.

## Figures and Tables

**Table 1:** The definition of characteristics for machine learning.

Characteristics	Definition
SN (present/absent)	Present if more than 5 consecutive beats were observed within any 30-s period during fixation-denied recording.
Latency before PN (present/absent)	Present if interval between positional change and nystagmus onset was more than 1 s.
Duration of PN (<1 min/≥1 min)	A positive finding was defined as PN lasting less than 1 min.
Pure vertical or vertical-component PN (present/absent)	Present if either pure vertical or vertical-component nystagmus was provoked during the Dix-Hallpike test or Roll Test.
The SPV_max_ of PN (°/s)	It was defined as the maximal SPV of either the vertical or horizontal component across all positional maneuvers.
Vertigo/dizziness during positional testing (present/absent)	Present if vertigo/ dizziness is provoked by the Dix-Hallpike test or Roll test.

PN, positional nystagmus; SN, spontaneous nystagmus; SPV_max_, Maximum slow phase velocity.

**Table 2:** Rationale for the selection of machine learning models.

Model	Rationale for Inclusion
Logistic Regression	This is a simple and foundational model in binary classification. Offers interpretability (Glassbox Model) and can serve as a useful comparison to more complex models.
Lasso Regression	It can reduce estimation variance while providing an interpretable final model. The smooth form of the constraint also offers greater stability than stepwise or best subset selection.
Random Forest	A reliable, work-horse algorithm that mitigates overfitting and provides global interpretability through average feature importance.
XGBoost	It is efficient at handling structured data and excels in capturing non-linear relationships and feature interactions, making it a suitable candidate for analyzing nystagmus features.
Support Vector Machine	They are widely used for classification, regression, and outlier detection, excelling in tasks like diagnosis and outcome prediction. They can construct hyperplanes that maximize the margin between classes, and employ kernel functions to handle complex, non-linearly separable data.

**Table 3:** Performance of machine learning models.

Models	AUC (95% CI)	Accuracy (95% CI)	Sensitivity (95% CI)	Specificity (95% CI)	PPV (95% CI)	NPV (95% CI)	F1 (95% CI)
Logistic Regression	0.9426 (0.9053–0.9742)	0.9250 (0.8938–0.9531)	0.8119 (0.7333–0.8889)	0.9772 (0.9541–0.9953)	0.9425 (0.8866–0.9875)	0.9185 (0.8797–0.9530)	0.8723 (0.8177–0.9215)
Lasso Regression	0.9405 (0.9009–0.9741)	0.9250 (0.8969–0.9531)	0.7822 (0.6979–0.8646)	0.9909 (0.9767–1.0000)	0.9753 (0.9351–1.0000)	0.9079 (0.8696–0.9422)	0.8681 (0.8092–0.9187)
Random Forest	0.9371 (0.8996–0.9690)	0.9219 (0.8906–0.9500)	0.8020 (0.7204–0.8727)	0.9772 (0.9569–0.9954)	0.9419 (0.8901–0.9875)	0.9145 (0.8776–0.9478)	0.8663 (0.8101–0.9149)
XGBoost	0.9427 (0.9047–0.9745)	0.9219 (0.8906–0.9500)	0.8119 (0.7292–0.8851)	0.9726 (0.9488–0.9911)	0.9318 (0.8736–0.9780)	0.9181 (0.8793–0.9516)	0.8677 (0.8125–0.9158)
Support Vector Machine	0.9392 (0.9001–0.9716)	0.9156 (0.8875–0.9438)	0.7822 (0.7010–0.8624)	0.9772 (0.9547–0.9955)	0.9405 (0.8817–0.9878)	0.9068 (0.8700–0.9417)	0.8541 (0.7957–0.9062)

AUC, area under the curve; PPV, positive predictive value; NPV, negative predictive value.

## Data Availability

The data supporting the findings of this study are available from the corresponding author on reasonable request.

## Supplementary Materials

The following supporting information can be downloaded at: https://www.mdpi.com/article/10.3390/diagnostics16213699/s1, Supplementary Figure S1: The tuning curves for all models; Supplementary Material S1: Logistic regression model for distinguishing VMwPV from PSC-BPPV and from HSC-BPPV; Supplementary Material S2: Model performance after removing vertigo/dizziness during positional testing; Supplementary Material S3: Model performance using three features (SN, SPV_max_ of PN, and pure vertical or vertical-component PN).

## Abbreviations

The following abbreviations are used in this manuscript:

AUC	Area under the curve
BPPV	Benign paroxysmal positional vertigo
FDR	False discovery rate
HSC-BPPV-can	Horizontal semicircular canal canalolithiasis
HSC-BPPV-cup	Horizontal semicircular canal cupulolithiasis
MD	Ménière’s disease
NPV	Negative predictive value
NU	Nodulus and uvula
PN	Positional nystagmus
PPV	Positive predictive value
PSC-BPPV	Posterior semicircular canal benign paroxysmal positional vertigo
PSN	Pseudo-spontaneous nystagmus
ROC	Receiver operating characteristic
SHAP	Shapley Additive Explanations
SN	Spontaneous nystagmus
SPV	Slow-phase velocity
SPV_max_	Maximum slow phase velocity
VM	Vestibular migraine
VMwPV	Vestibular migraine with positional vertigo
