# Supplementary material for: Ranking Features Distinguish Vestibular Migraine with Positional Vertigo from Benign Paroxysmal Positional Vertigo Using Machine Learning
Source: Diagnostics (Basel). 2026 Nov 9;16(21):3699. doi: 10.3390/diagnostics16213699 (PMC13762269; doi:10.3390/diagnostics16213699)
Supplement: Supplementary file 1 [file diagnostics-16-03699-s001.zip › Supplementary Figure S1.pdf]

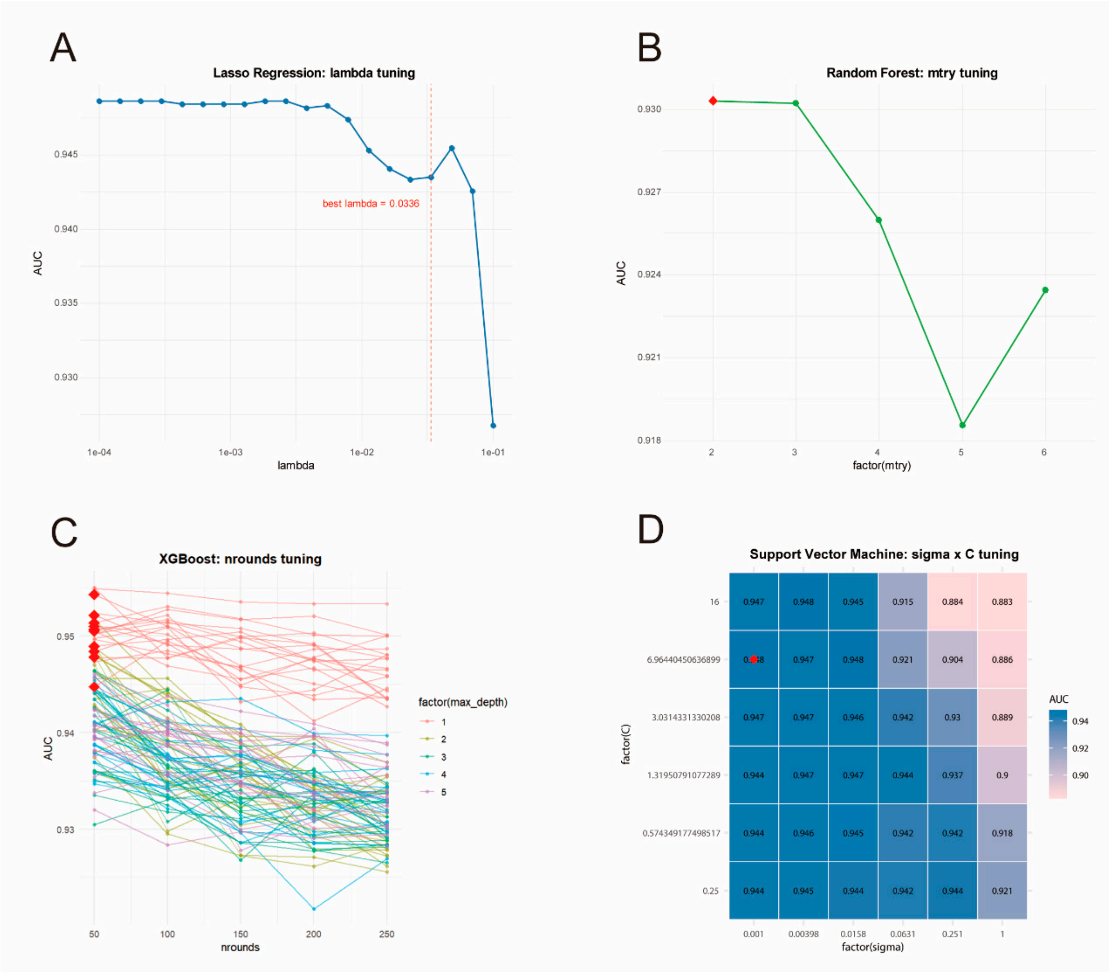

Supplementary Figure S1. The tuning curves for Lasso regression(A), Random Forest(B), XGBoost (C) and Support Vector Machine (D).
