# Supplementary material for: Ranking Features Distinguish Vestibular Migraine with Positional Vertigo from Benign Paroxysmal Positional Vertigo Using Machine Learning
Source: Diagnostics (Basel). 2026 Nov 9;16(21):3699. doi: 10.3390/diagnostics16213699 (PMC13762269; doi:10.3390/diagnostics16213699)
Supplement: Supplementary file 1 [file diagnostics-16-03699-s001.zip › Supplementary material S1.pdf]

Logistic Regression Model for Distinguishing VMwPV from PSC-BPPV and from HSC-BPPV

As shown in Table 1, in the subgroup analysis distinguishing VMwPV (n = 101) from PSC-BPPV (n = 159), the Logistic Regression model achieved an AUC of 0.9365 (0.8918 - 0.9717). In the subgroup analysis distinguishing VMwPV (n = 101) from HSC-BPPV (n = 60), the model achieved an AUC of 0.9554 (0.9231 - 0.9811).

Table 1. Performance of the Logistic Regression model in subgroup analyses distinguishing VMwPV from PSC-BPPV and from HSC-BPPV using repeated stratified 5-fold cross-validation (10 repeats).

Groups	AUC (95%CI)	Accuracy (95%CI)	Sensitivity (95%CI)	Specificity (95%CI)	PPV (95%CI)	NPV (95%CI)	F1 (95%CI)
VMwPV vs PSC-BPPV	0.9365 (0.8918 - 0.9717)	0.9269 (0.8923 - 0.9577)	0.8614 (0.7884 - 0.9255)	0.9686 (0.9404 - 0.9936)	0.9457 (0.8974 - 0.9885)	0.9167 (0.8706 - 0.9573)	0.9016 (0.8528 - 0.9432)
VMwPV vs HSC-BPPV	0.9554 (0.9231 - 0.9811)	0.8944 (0.8447 - 0.9379)	0.8911 (0.8242 - 0.9490)	0.9000 (0.8197 - 0.9697)	0.9375 (0.8842 - 0.9804)	0.8308 (0.7324 - 0.9167)	0.9137 (0.8687 - 0.9519)

VMwPV, vestibular migraine with positional vertigo; BPPV, benign paroxysmal positional vertigo; PSC-BPPV, Posterior semicircular canal BPPV; HSC-BPPV, Horizontal semicircular canal BPPV; AUC, area under the curve; PPV, positive predictive value; NPV, negative predictive Value.
