# Supplementary material for: Ranking Features Distinguish Vestibular Migraine with Positional Vertigo from Benign Paroxysmal Positional Vertigo Using Machine Learning
Source: Diagnostics (Basel). 2026 Nov 9;16(21):3699. doi: 10.3390/diagnostics16213699 (PMC13762269; doi:10.3390/diagnostics16213699)
Supplement: Supplementary file 1 [file diagnostics-16-03699-s001.zip › Supplementary material S2.pdf]

Model Performance After Removing Vertigo/dizziness During Positional Testing

Since vertigo/dizziness during positional testing was reported in all BPPV patients but in only 30.7% of VMwPV patients, we removed this variable and repeated the machine learning analysis. Hyperparameter tuning was performed within a nested cross-validation framework (Figure 1). As shown in Table 1 and Figure 2A, all model performance metrics (AUC, accuracy, sensitivity, specificity, PPV, NPV, and F1) were summarized as mean with 95% confidence intervals across 50 outer validation folds. All models retained AUC > 0.90.

After removing "vertigo/dizziness during positional testing", the DeLong comparison showed that Logistic Regression and Lasso Regression had significantly higher AUC than Support Vector Machine (FDR-adjusted p = 0.02 and 0.03, respectively), whereas no other pairwise comparisons reached statistical significance (Table 2).

Brier scores of Logistic Regression, Lasso Regression, Random Forest, XGBoost and Support Vector Machine were 0.0785, 0.0806, 0.0834, 0.0801 and 0.0914, respectively. Compared with those models that contain vertigo/dizziness during positional testing, the Brier scores of models increased only modestly (all still < 0.1), indicating preserved calibration. As shown in Figure 2B, all models continued to provide higher net benefit than the "treat all" and "treat none" strategies, indicating preserved clinical utility. As shown in Figure 2C, calibration curves were constructed using the same out-of-fold predictions from repeated stratified 5-fold cross-validation (10 repeats). It showed that all models closely followed the ideal line (y = x) across the probability range.

As for the result of SHAP analysis, PN lasting < 1min remained the leading predictor (Figure 3).

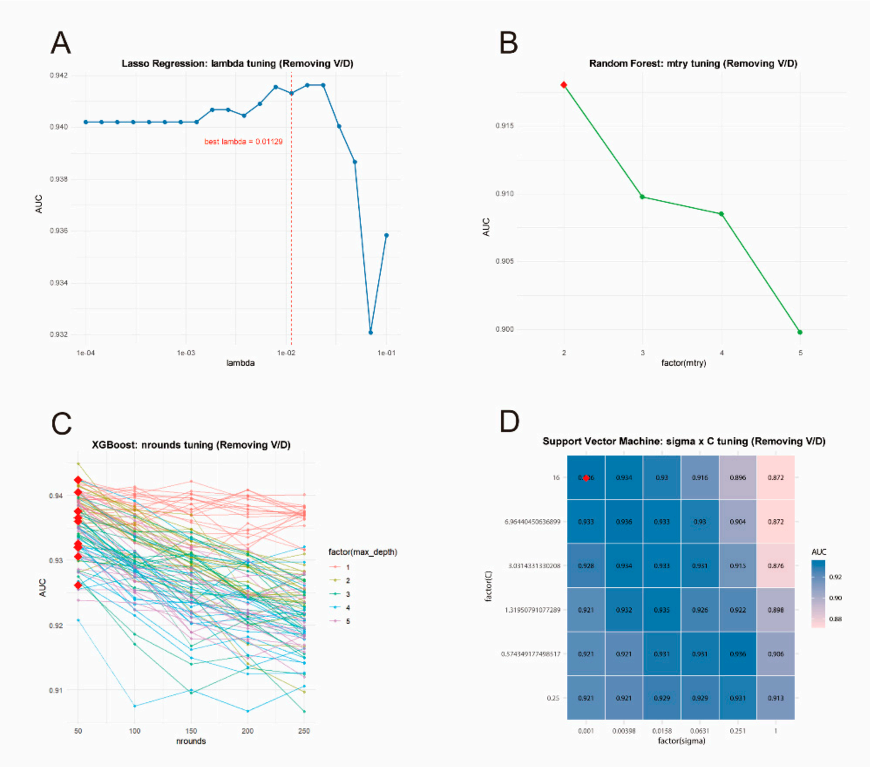

Figure 1. The tuning curves for Lasso Regression (A), Random Forest (B), XGBoost (C) and Support Vector Machine (D).

Table 1. The performance of models

Models	AUC (95% CI)	Accuracy (95% CI)	Sensitivity (95% CI)	Specificity (95% CI)	PPV (95% CI)	NPV (95% CI)	F1 (95% CI)
Logistic regression	0.9350 (0.8990 - 0.9646)	0.8938 (0.8562 - 0.9250)	0.7921 (0.7091 - 0.8654)	0.9406 (0.9074 - 0.9707)	0.8602 (0.7879 - 0.9286)	0.9075 (0.8667 - 0.9420)	0.8247 (0.7636 - 0.8768)
Lasso Regression	0.9326 (0.8968 - 0.9643)	0.8938 (0.8594 - 0.9281)	0.7822 (0.6979 - 0.8617)	0.9452 (0.9155 - 0.9731)	0.8681 (0.7979 - 0.9348)	0.9039 (0.8645 - 0.9409)	0.8229 (0.7630 - 0.8792)
Random Forest	0.9235 (0.8833 - 0.9582)	0.8906 (0.8562 - 0.9219)	0.7525 (0.6695 - 0.8349)	0.9543 (0.9238 - 0.9787)	0.8837 (0.8090 - 0.9467)	0.8932 (0.8534 - 0.9301)	0.8128 (0.7470 - 0.8677)
XGBoost	0.9313 (0.8954 - 0.9627)	0.8875 (0.8500 - 0.9219)	0.7624 (0.6729 - 0.8447)	0.9452 (0.9124 - 0.9737)	0.8652 (0.7889 - 0.9320)	0.8961 (0.8565 - 0.9359)	0.8105 (0.7432 - 0.8692)
Support Vector Machine	0.9225 (0.8821 - 0.9565)	0.8750 (0.8406 - 0.9125)	0.7525 (0.6698 - 0.8351)	0.9315 (0.8952 - 0.9636)	0.8352 (0.7576 - 0.9136)	0.8908 (0.8500 - 0.9306)	0.7917 (0.7253 - 0.8529)

AUC, area under the curve; PPV, positive predictive value; NPV, negative Predictive Value.

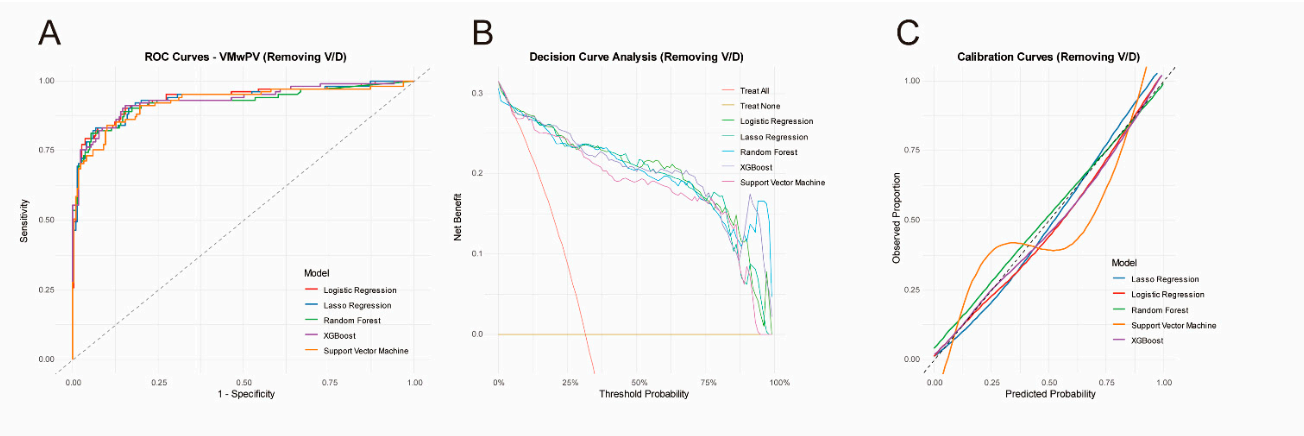

Figure 2. The performance of all models after removing vertigo/dizziness(V/D) during positional testing. The ROC curve of Logistic regression, Lasso Regression, Random Forest, XGBoost and Support Vector Machine (A), the Decision curve analysis (B), and the Calibration curves (C) of all models

Table 2. FDR-adjusted p-value matrix for pairwise DeLong comparisons of AUC between models

Models	Logistic regression	Lasso Regression	Random Forest	XGBoost	Support vector machine
Logistic Regression	NA	0.3564	0.3564	0.6436	0.02153
Lasso Regression	0.35640	NA	0.3564	0.9019	0.03010
Random Forest	0.35640	0.3564	NA	0.3564	0.90190
XGBoost	0.64360	0.9019	0.3564	NA	0.35640
Support Vector Machine	0.02153	0.0301	0.9019	0.3564	NA

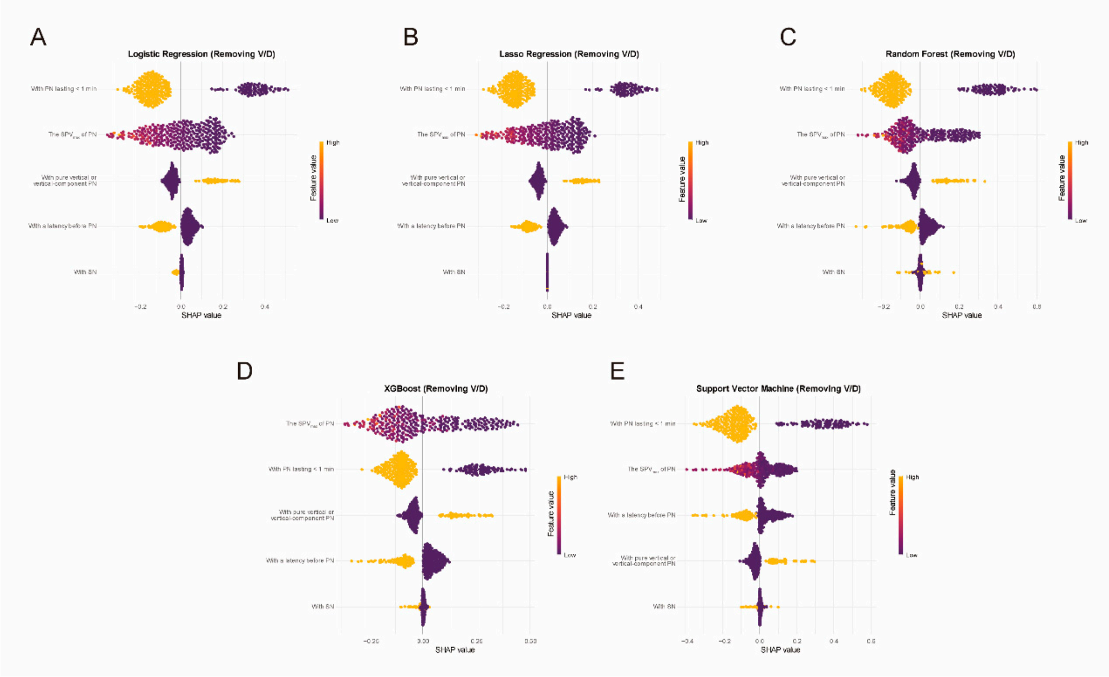

Figure 3. SHAP feature importance beeswarm plots of five models after removing vertigo/dizziness(V/D) during positional testing: (A) Logistic Regression, (B) Lasso Regression, (C) Random Forest, (D) XGBoost, and (E) Support Vector Machine. SHAP values, with the top-ranked features being the most influential predictors across models. VMwPV, vestibular migraine with positional vertigo; PN, positional nystagmus; SN, spontaneous nystagmus; SPV_{max}, maximum slow phase velocity; SHAP, Shapley Additive Explanations.
