# Supplementary material for: Ranking Features Distinguish Vestibular Migraine with Positional Vertigo from Benign Paroxysmal Positional Vertigo Using Machine Learning
Source: Diagnostics (Basel). 2026 Nov 9;16(21):3699. doi: 10.3390/diagnostics16213699 (PMC13762269; doi:10.3390/diagnostics16213699)
Supplement: Supplementary file 1 [file diagnostics-16-03699-s001.zip › Supplementary material S3.pdf]

Model Performance Using Three Features (SN, SPVmax of PN, and Pure Vertical or Vertical-Component PN)

We further analyzed models using only features outside the BPPV diagnostic criteria: SN, SPV_{max} of PN, and pure vertical or vertical-component PN. Hyperparameter tuning was performed within a nested cross-validation framework (Figure 1). As shown in Table 1 and Figure 2A, all model performance metrics (AUC, accuracy, sensitivity, specificity, PPV, NPV, and F1) were summarized as mean with 95% confidence intervals across 50 outer validation folds.

The DeLong comparison showed no statistically significant differences among Logistic Regression, Lasso Regression, Random Forest, and XGBoost (all FDR-adjusted $p > 0.05$). However, Support Vector Machine had a significantly lower AUC compared to Logistic Regression, Lasso Regression and XGBoost (all FDR-adjusted $p < 0.05$) (Table 2).

Brier scores of Logistic Regression, Lasso Regression, Random Forest, XGBoost and Support Vector Machine were 0.1369, 0.1424, 0.1329, 0.1220, and 0.1552, respectively. As shown in Figure 2B, all models continued to provide higher net benefit than the "treat all" and "treat none" strategies, indicating preserved clinical utility. Calibration curves were constructed using the same out-of-fold predictions from repeated stratified 5-fold cross-validation (10 repeats), and the resulting curves generally followed the ideal line (Figure 2C).

As for the SHAP analysis, the feature importance ranking among the three objective variables was largely consistent across models, with SPV_{max} of PN and pure vertical or vertical-component PN ranking as the top two predictors (Figure 3).

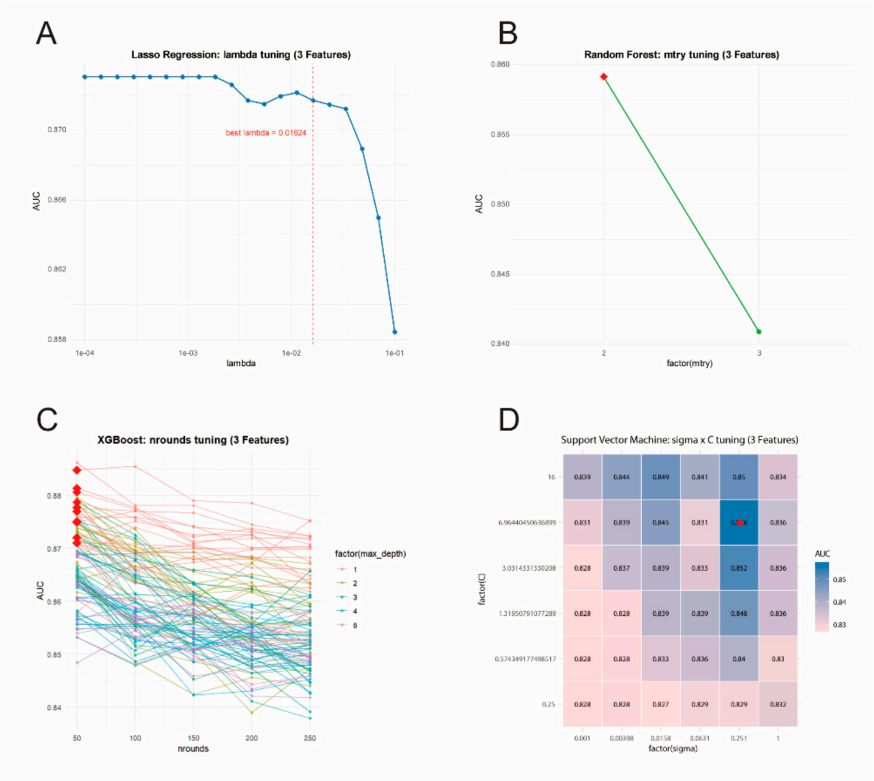

Figure 1. The tuning curves for Lasso Regression (A), Random Forest (B), XGBoost (C) and Support Vector Machine (D).

Table 1. The performance of models

Models	AUC (95% CI)	Accuracy (95% CI)	Sensitivity (95% CI)	Specificity (95% CI)	PPV (95% CI)	NPV (95% CI)	F1 (95% CI)
Logistic Regression	0.8560 (0.8092 - 0.8979)	0.7844 (0.7375 - 0.8281)	0.5248 (0.4242 - 0.6250)	0.9041 (0.8640 - 0.9412)	0.7162 (0.6027 - 0.8171)	0.8049 (0.7579 - 0.8531)	0.6057 (0.5106 - 0.6901)
Lasso Regression	0.8583 (0.8118 - 0.8999)	0.7906 (0.7469 - 0.8344)	0.5050 (0.4000 - 0.6000)	0.9224 (0.8844 - 0.9565)	0.7500 (0.6393 - 0.8530)	0.8016 (0.7542 - 0.8488)	0.6036 (0.5068 - 0.6861)
Random Forest	0.8653 (0.8173 - 0.9048)	0.8281 (0.7844 - 0.8657)	0.6535 (0.5588 - 0.7476)	0.9087 (0.8688 - 0.9445)	0.7674 (0.6790 - 0.8519)	0.8504 (0.8026 - 0.8938)	0.7059 (0.6264 - 0.7738)
XGBoost	0.8732 (0.8277 - 0.9150)	0.8531 (0.8125 - 0.8906)	0.6832 (0.5887 - 0.7755)	0.9315 (0.8979 - 0.9631)	0.8214 (0.7375 - 0.8989)	0.8644 (0.8182 - 0.9076)	0.7459 (0.6705 - 0.8103)
Support Vector Machine	0.8313 (0.7814 - 0.8802)	0.7906 (0.7438 - 0.8344)	0.4950 (0.3958 - 0.5957)	0.9269 (0.8905 - 0.9595)	0.7576 (0.6533 - 0.8571)	0.7992 (0.7480 - 0.8490)	0.5988 (0.5063 - 0.6860)

AUC, area under the curve; PPV, positive predictive value; NPV, negative Predictive Value.

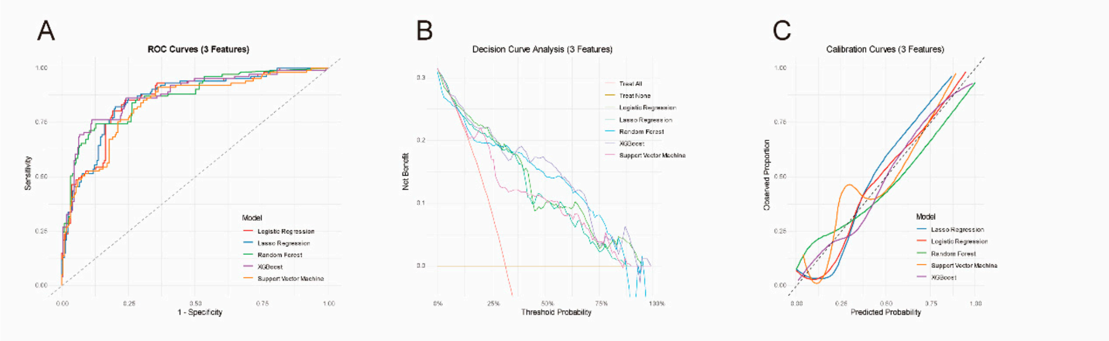

Figure 2. The performance of all models after removing vertigo/dizziness(V/D) during positional testing. The ROC curve of Logistic Regression, Lasso Regression, Random Forest XGBoost and Support Vector Machine (A), the Decision curve analysis (B), and the Calibration curves (C) of all models

Table 2. FDR-adjusted p-value matrix for pairwise DeLong comparisons of AUC between models

Models	Logistic regression	Lasso Regression	Random Forest	XGBoost	Support vector machine
Logistic Regression	NA	0.49640	0.60590	0.31430	0.02079
Lasso Regression	0.49640	NA	0.65130	0.38060	0.01263
Random Forest	0.60590	0.65130	NA	0.45780	0.08882
XGBoost	0.31430	0.38060	0.45780	NA	0.01263
Support Vector Machine	0.02079	0.01263	0.08882	0.01263	NA

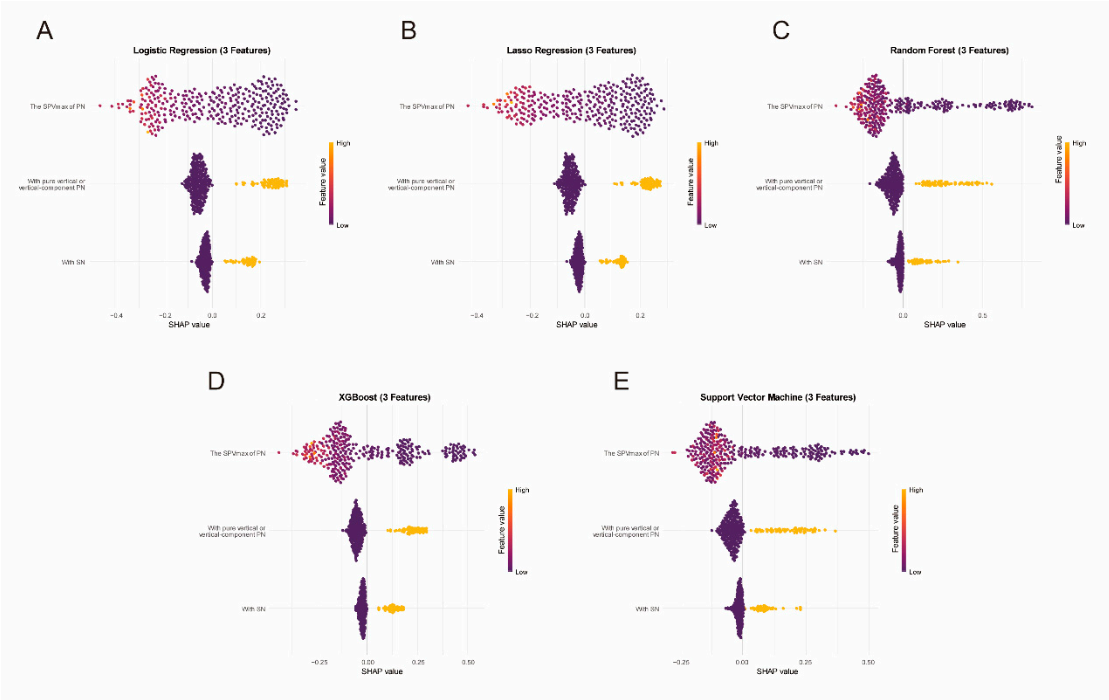

Figure 3. SHAP feature importance beeswarm plots of five models after removing vertigo/dizziness(V/D) during positional testing: (A) Logistic Regression, (B) Lasso Regression, (C) Random Forest, (D) XGBoost, and (E) Support Vector Machine. SHAP values, with the top-ranked features being the most influential predictors across models. VMwPV, vestibular migraine with positional vertigo; PN, positional nystagmus; SN, spontaneous nystagmus; SPV_{max}, maximum slow phase velocity; SHAP, Shapley Additive Explanations.
